# Ciliary photoreceptors in sea urchin larvae indicate pan-deuterostome cell type conservation

**DOI:** 10.1186/s12915-021-01194-y

**Published:** 2021-12-04

**Authors:** Jonathan E. Valencia, Roberto Feuda, Dan O. Mellott, Robert D. Burke, Isabelle S. Peter

**Affiliations:** 1 Division of Biology and Biological Engineering, California Institute of Technology, Pasadena, CA 91125 USA; 2 Present address: Department of Genetics and Genome Biology, University of Leicester, Leicester, UK; 3 Department of Biochemistry and Microbiology, University of Victoria, Victoria, British Columbia Canada

**Keywords:** Cell type evolution, Photoreceptors, Regulatory state, GRN evolution, Echinoderms

## Abstract

**Background:**

The evolutionary history of cell types provides insights into how morphological and functional complexity arose during animal evolution. Photoreceptor cell types are particularly broadly distributed throughout Bilateria; however, their evolutionary relationship is so far unresolved. Previous studies indicate that ciliary photoreceptors are homologous at least within chordates, and here, we present evidence that a related form of this cell type is also present in echinoderm larvae.

**Results:**

Larvae of the purple sea urchin *Strongylocentrotus purpuratus* have photoreceptors that are positioned bilaterally in the oral/anterior apical neurogenic ectoderm. Here, we show that these photoreceptors express the transcription factor Rx, which is commonly expressed in ciliary photoreceptors, together with an atypical opsin of the G_O_ family, opsin3.2, which localizes in particular to the cilia on the cell surface of photoreceptors. We show that these ciliary photoreceptors express the neuronal marker synaptotagmin and are located in proximity to pigment cells. Furthermore, we systematically identified additional transcription factors expressed in these larval photoreceptors and found that a majority are orthologous to transcription factors expressed in vertebrate ciliary photoreceptors, including Otx, Six3, Tbx2/3, and Rx. Based on the developmental expression of *rx*, these photoreceptors derive from the anterior apical neurogenic ectoderm. However, genes typically involved in eye development in bilateria, including *pax6*, *six1/2*, *eya*, and *dac*, are not expressed in sea urchin larval photoreceptors but are instead co-expressed in the hydropore canal.

**Conclusions:**

Based on transcription factor expression, location, and developmental origin, we conclude that the sea urchin larval photoreceptors constitute a cell type that is likely homologous to the ciliary photoreceptors present in chordates.

**Supplementary Information:**

The online version contains supplementary material available at 10.1186/s12915-021-01194-y.

## Background

The remarkable similarity among certain cell types in distantly related animals suggests that some cell types have been present as a functional unit for a very long time during animal evolution [1–3]. The similarity between these cell types is reflected in specific structural and functional properties and also at the molecular level in the expression of similar gene sets. In particular, the expression of similar combinations of transcription factors appears to be one of the signatures of cell type conservation [2]. Resolving the evolutionary ancestry of cell types, and identifying the changes that have occurred during cell type evolution at the structural, molecular, and regulatory level, promises to reveal insights into the evolutionary processes that contributed to the definition of diverse cell types in extant animals.

Photoreceptor cells represent a prominent example of a cell type that is broadly shared among metazoans [4]. However, despite extensive research, the evolutionary history of photoreceptor cell types remains unclear [2, 4–7]. Two classes of photoreceptors are commonly encountered in bilateria, ciliary photoreceptors, which are predominantly deployed in deuterostomes, and rhabdomeric photoreceptors, which are typically present in protostomes [8, 9]. Recent observations indicate that both classes of photoreceptors are more broadly distributed within the bilateria. Thus, ciliary photoreceptors are also present outside of deuterostomes, for example, in the annelid *Platynereis dumerilii* [9] and in the cubozoan jellyfish *Triedalia cystophora* [10], while rhabdomeric photoreceptors are present in deuterostomes including sea urchins and amphioxus [11–13]. Ciliary and rhabdomeric photoreceptors are morphologically distinct by possessing different cell surface extensions that increase the photosensitive area. They are also molecularly distinct by deploying different pathways for transducing the intracellular response to photo-excitement [14]. These molecular differences suggest that the two types of photoreceptors are non-homologous although they most likely co-existed in bilaterian ancestors [14]. On the other hand, the expression of Pax6 and other transcription factors of the retinal determination network during early development of eyes irrespective of the photoreceptor cell type suggests similarities that could be explained by descent from a common proto-eye [15, 16]. Thus, there is ambiguous evidence that leaves unresolved whether photoreceptors arose just once and subsequently evolved into the different variations of photoreceptors or whether photoreceptor cells evolved several times independently. Data from different clades across the phylogeny are therefore required to resolve the evolutionary history of photoreceptor cell types within bilateria.

Within vertebrates, comparative analyses indicate that ciliary photoreceptors and eyes show considerable similarity [17, 18]. At the morphological level, vertebrate eyes have a distinct position within the neuroectoderm, anterior and bilateral, and include rod and cone ciliary photoreceptors as well as pigment cells [19]. At the developmental level, eyes and photoreceptors derive from comparable embryological origins within vertebrates, the anterior neuroectoderm [20]. Furthermore, ciliary photoreceptors in vertebrate eyes show similarities at the molecular level, expressing a similar type of c-opsin and a similar set of transcription factors including Otx, Rx, and Crx [21–27]. The transcription factor Rx plays an important role in vertebrate eye development [24]. During early eye development, Rx regulates the expression of *pax6* and *six3*, while in differentiated ciliary photoreceptors, Rx regulates the expression of *opsin* and other effector genes [25, 27–29]. Rx is also expressed in ciliary photoreceptors of non-vertebrate chordates, including amphioxus [11] and *Ciona intestinalis* [30], and in the ciliary photoreceptors of protostome marine annelids [9], indicating that Rx is involved in the specification of ciliary photoreceptors throughout bilateria.

Further structural and molecular similarities were observed between the ciliary photoreceptors and pigment cells of the frontal eye in amphioxus and the ciliary photoreceptors and pigment cells of vertebrate eyes [11]. These observations suggest that the ciliary photoreceptors in the frontal eye of amphioxus are homologous to the ciliary photoreceptors in vertebrate eyes [2, 11]. Within chordates, ciliary photoreceptors are therefore not just the predominant cell type of the eye but also very likely constitute a homologous cell type as defined by descent from a common ancestral photoreceptor cell type. It remains unclear however whether ciliary photoreceptors are also present in extant non-chordate deuterostomes, and if so, whether these cells share sufficient similarities with chordate photoreceptors to indicate that ciliary photoreceptors are homologous throughout deuterostomes and thus present in the last common ancestor of deuterostomes.

In the sister clade of chordates, the ambulacraria that includes hemichordates and echinoderms, rhabdomeric photoreceptors were found in the larval eyes of enteropneusts and in the tube feet of adult sea urchins [13, 14, 31]. Furthermore, the c-opsin *opsin1* is expressed in several tissues of adult sea urchins and in other echinoderms [32]. In sea urchin larvae, the expression of the Go-opsin *opsin3.2* was detected in cells that were described as non-directional photoreceptors [33], and opsin3.2 was shown to be involved in the behavioral response to light [34]. These studies suggest that functional photoreceptor cells are present in sea urchin larvae, although the cell type identity of these photoreceptors has not been established.

Here, we present evidence indicating that opsin expressing cells in the larvae of the purple sea urchin *Strongylocentrotus purpuratus* correspond to ciliary photoreceptors. We show that *opsin3.2* is co-expressed with Rx and with synaptotagmin in bilateral clusters of cells in the oral/anterior apical neurogenic ectoderm from which the photoreceptors originate developmentally. On the cell surface of these photoreceptors, Opsin co-localizes with tubulin-containing cilia, indicating that they serve as a photosensitive structure. Pigment cells that might contribute to an oral/aboral bias in light perception were found to be located near the photoreceptor cells. In addition to Rx, sea urchin photoreceptors express several transcription factors including Otx and Six3, but not Pax6, that indicate similarity to the regulatory state of vertebrate ciliary photoreceptors. Our findings thus suggest that sea urchin larvae possess ciliary photoreceptors that are related to the ciliary photoreceptors present in chordates.

## Results

### *Rx* and *opsin3.2* are co-expressed in bilateral clusters of cells

**Fig. 1:**
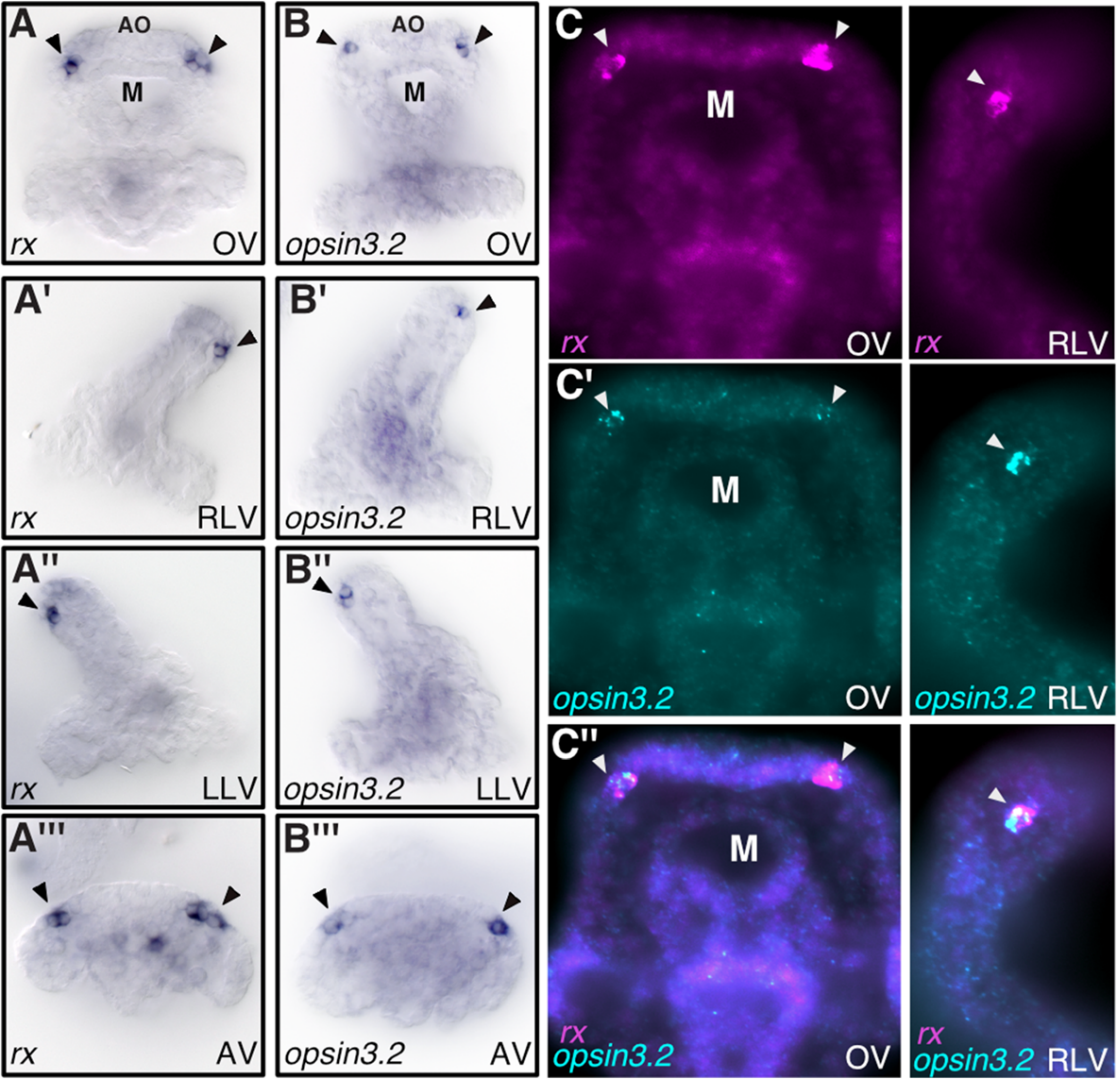
Spatial expression of *rx* and *opsin 3.2* in photoreceptors of sea urchin larvae. **A**, **A’**, **A”**, **A”’** WMISH showing the expression of *rx* on the oral side of the apical organ at 72 h. **B**, **B’**, **B”**, **B”’** WMISH for *opsin3.2* expression. **C**, **C’**, **C”** Double-fluorescent WMISH for *rx* (magenta, **C**) and *opsin3.2* (green, **C’**), overlay shown in **C”**. Arrowhead indicates photoreceptors. M, mouth; AO, apical organ; OV, oral view; RLV, right lateral view; LLV, left lateral view; AV, apical view

The transcription factor Rx is expressed in ciliary photoreceptors of chordates as well as protostomes. When expression of *rx* was examined in sea urchin larvae by whole-mount in situ hybridization (WMISH), expression was found to be restricted to bilateral clusters of 1–3 cells located on the oral side of the neurogenic apical ectoderm at 72 h after fertilization (Fig. 1A). The particular location of *rx*-expressing cells at the anterior periphery of the nervous system and above the mouth suggested that these cells might correspond to photoreceptor cells.

To determine if the putative larval photoreceptor cells express any type of opsin, we analyzed available transcriptome data during sea urchin development [35]. Eight opsin genes are encoded in the sea urchin genome, but only two of these, *opsin2* and *opsin3.2*, showed expression at the larval stage (72 h), while transcripts of other opsin genes were not detectable during early development (Fig. S1A). To determine whether these *opsin* genes are expressed in putative photoreceptors, we analyzed the spatial expression of *opsin2* and *opsin3.2* by WMISH. The results show that *opsin2* is expressed in the stomodeum (mouth) region and part of the ciliary band between the lower arms (Fig. S1B). On the other hand, *opsin 3.2* is expressed in bilateral clusters of cells on the oral side of the apical neurogenic ectoderm, consistent with previous results (Fig. 1B and Fig. S1B) [33]. To determine whether *rx* and *opsin3.2* are co-expressed, we performed double fluorescence WMISH. The results show that *rx* and *opsin3.2* are indeed co-expressed in the putative photoreceptor cells (Fig. 1C).

To clarify the phylogenetic affinity of opsin3.2, we performed a phylogenetic analysis of metazoans opsins. A set of 242 opsins genes was identified using a combination of BLASTP and motif annotation, and the phylogenetic relationships were determined using Bayesian and maximum likelihood inference. The results indicate that opsin3.2 is part of a monophyletic group of Go opsins, consistent with previous analyses (Fig. S2) [36]. This class of opsins represents atypical opsins that are expressed in ciliary photoreceptors of scallops [36–38] and in rhabdomeric photoreceptors in *Platynereis* [39].

### Expression of synaptotagmin indicates neural identity of photoreceptors

**Fig. 2:**
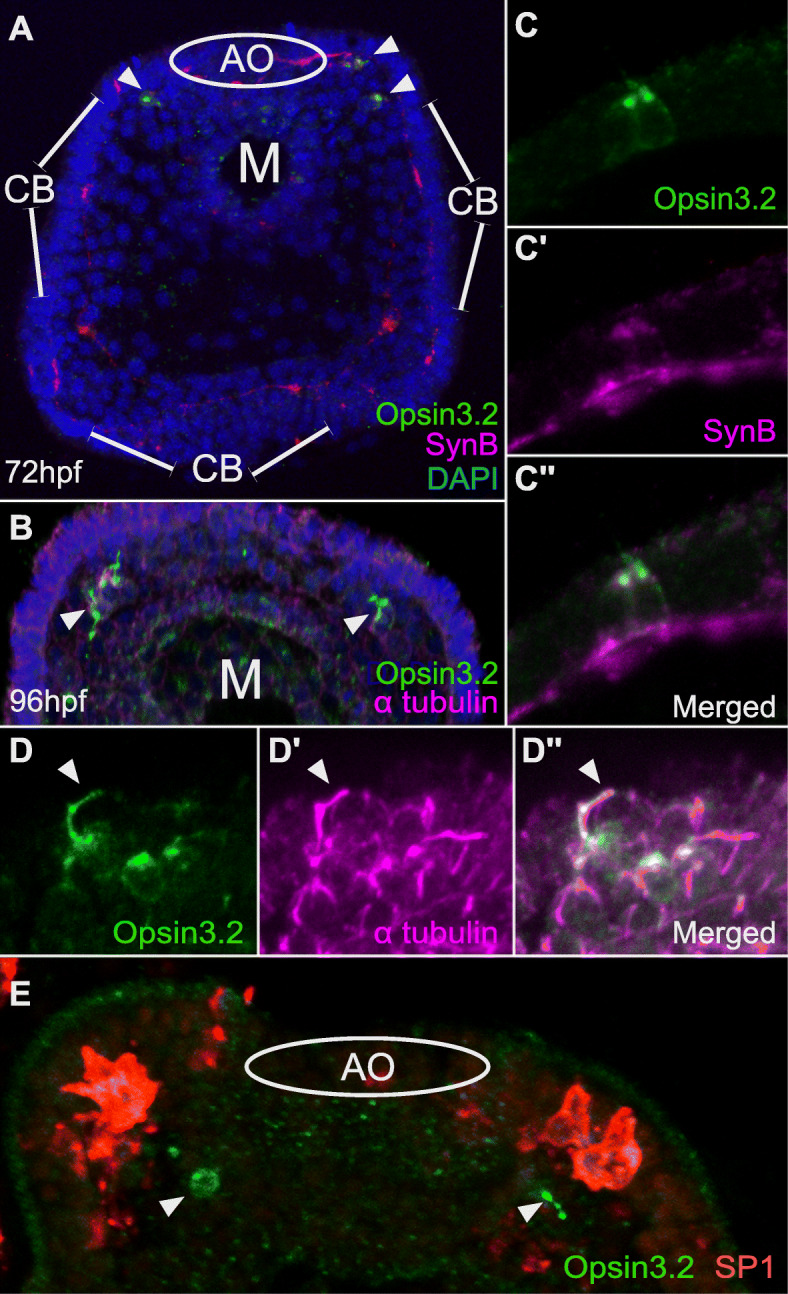
Immunostaining showing the expression of opsin 3.2 in neuronal ciliary photoreceptors. Confocal laser scanning images of whole-mount *S. purpuratus* larvae showing immunostaining **A** of opsin 3.2 and synaptotagmin B at 72 h and **B** of opsin3.2 and α-tubulin at 96 h. **C** Confocal images of co-immunostaining for opsin3.2 (green, **C**) and synaptotagmin B (magenta, **C’**), showing co-expression (**C”**). **D** Confocal images of co-immunostaining for opsin3.2 with α-tubulin (**D’**) showing co-localization in cell surface cilia of photoreceptors (**D”**). **E** Co-immunostaining of opsin3.2 (green) and pigment cell-specific SP1 (red) showing pigment cells located in proximity to photoreceptors. Arrowheads indicate photoreceptors (**A**, **B**, **E**) and cilia on photoreceptors (**D**, **D’**, **D”**). M, mouth; AO, apical organ; CB, ciliated band

Typically, the response to light is transmitted from photoreceptors to the nervous system through synaptic exocytosis of neurotransmitters [40]. In sea urchin larvae, the bilateral clusters of *opsin3.2*-expressing cells are positioned at the periphery of the anterior neurogenic domain, which becomes the apical organ. To determine if the putative photoreceptor cells correspond to neurons, we analyzed the expression of synaptotagmin, a pre-synaptic neuronal protein involved in synaptic vesicle exocytosis [40–42]. Immunostaining was performed with antibodies against the pan-neuronal synaptotagmin B [43] and with rat polyclonal antibodies against sea urchin opsin3.2 in 72 h sea urchin larvae. Expression of opsin3.2 protein was detected in bilateral clusters of cells, similar to the localization of *opsin3.2* mRNA (Fig. 2A, B). The results show that opsin3.2 is expressed in a subset of synaptotagmin B-expressing cells (Fig. 2A, C). This result indicates that the putative photoreceptor cells are neuronal and thus may be capable of transmitting a signal in response to light stimulation. Synaptotagmin-containing axons of the photoreceptors indeed were found to project to the neuropil underlying the apical organ (Fig. S3A). In comparison, co-immunostaining of opsin3.2 and serotonin showed expression in separate cells, indicating that, as expected, the photoreceptor cells are not serotonergic neurons (Fig. S3B).

### Presence of opsin in cilia of photoreceptors

Ciliary and rhabdomeric photoreceptor cells possess morphologically distinct cell surface structures, cilia and rhabdomeres, respectively, that serve as extended photosensitive areas for the presentation of opsins [44]. Cilia and rhabdomeres are not just structurally distinct, they are also composed of distinct molecular components. Cilia have a core of microtubules whereas rhabdomeres consist of numerous microvilli with a core of actin filaments. To test whether the larval photoreceptors in sea urchins belong to the class of ciliary photoreceptors, we thus analyzed whether these cells possess ciliary cell surface extensions where opsin proteins accumulate. We used antibodies against α-tubulin to detect the presence of microtubules in photoreceptor cells by immunostaining. The results show that α-tubulin is present in the cilia of cells within the apical and ciliated band neurogenic ectoderm (Fig. 2B, D). Co-immunostaining with antibodies against opsin3.2 showed that while opsin3.2 is present throughout the cell body, it co-localizes in particular with α-tubulin in short cilia on the apices of photoreceptor cells (Fig. 2B, D). The accumulation of opsins in the cilia of photoreceptor cells suggests that these cilia serve as photosensitive cell surface structures, indicating that the sea urchin larval photoreceptors belong to the class of ciliary photoreceptors.

### Ciliary photoreceptors are associated with pigment cells

In order to enable a directional perception of light, photoreceptors are typically associated with shading pigments [14, 44]. A previous study did not find evidence for specialized shading pigments in cells of the apical organ and the adjacent ciliary band when assessed by electron microscopy [33]. However, sea urchin larvae possess immune cells that contain echinochrome pigments, and these pigment cells are broadly distributed within the aboral ectoderm [45]. Since echinochrome pigments are usually no longer visible once embryos are fixed, these pigment cells would have remained undetected in the previous study. To determine if pigment cells are present near the photoreceptors in sea urchin larvae, we performed immunostaining using SP1 antibodies that detect pigment cells [46]. Indeed, pigment cells were found within 2–3 cell diameters of the photoreceptors, embedded in the aboral ectodermal epithelium adjacent to the apical organ (Fig. 2E). Although the pigment cells are not in immediate contact with the photoreceptor cells, they are still potentially close enough to provide shading and enable directional photoreception. In addition, pigment cells are dispersed throughout the aboral ectoderm, but entirely absent from the oral ectoderm, creating a potential bias in the intensity of light perceived from the oral versus aboral side of the larva.

### Transcription factor expression and developmental origin of ciliary photoreceptors

**Fig. 3:**
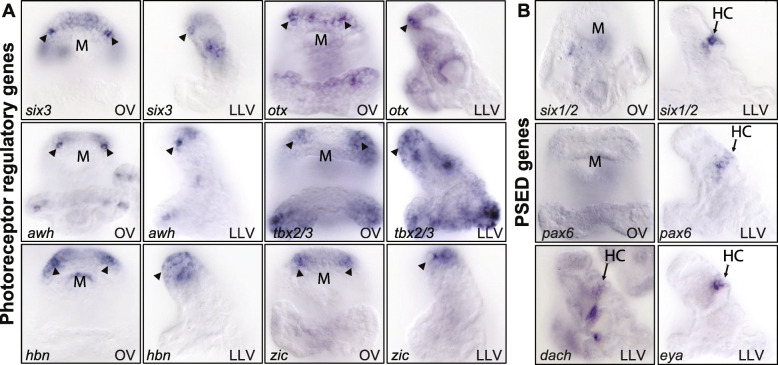
Expression of photoreceptor regulatory state and PSED genes in sea urchin larvae. **A** WMISH in 72-h larvae showing the expression of selected regulatory genes in ciliary photoreceptors (arrowheads). Additional regulatory genes expressed in photoreceptors are shown in Fig. 4. **B** WMISH in 72-h larvae showing the expression of *pax6*, *six1/2*, *dach*, and *eya* in the hydropore canal (HC). OV, oral view; LLV, left lateral view

**Fig. 4:**
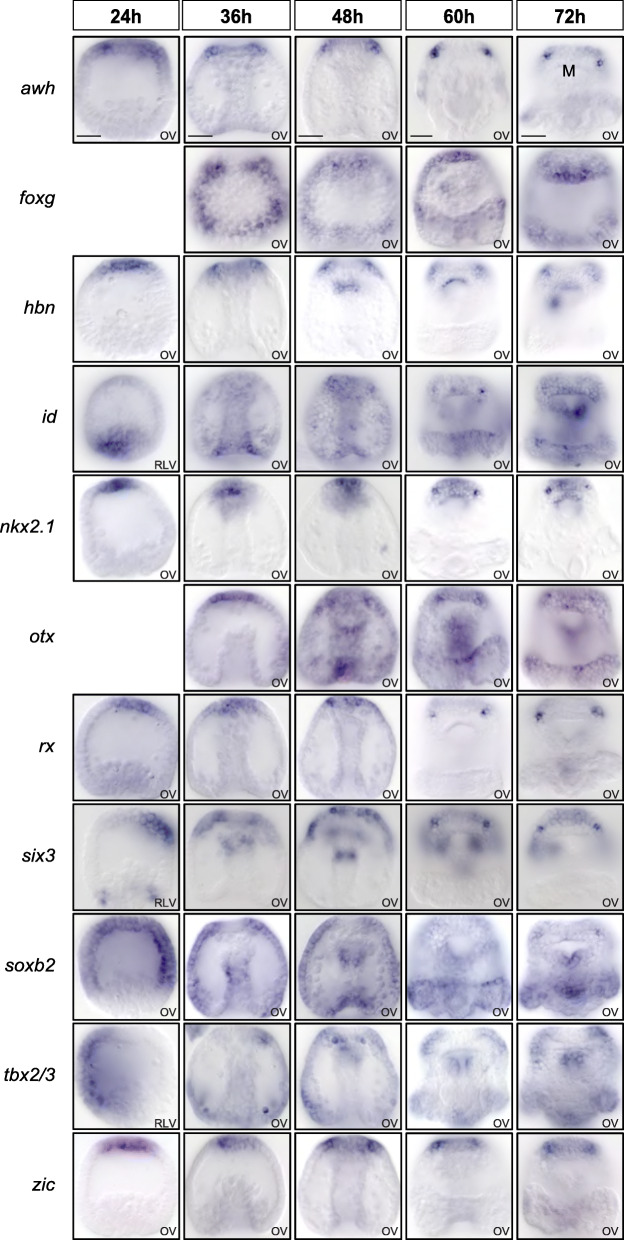
Time course of regulatory gene expression during photoreceptor development. Embryos were stained by WMISH with probes detecting the expression of regulatory genes for which expression was observed in larval photoreceptor cells at 60 and/or 72 h. Developmental stages from pregastrula (24 h) to pluteus larva (72 h) are indicated. OV, oral view; RLV, right lateral view. Scale bars 20 μm

In a genome-wide study of transcription factor expression in sea urchins, to be reported elsewhere, several additional genes encoding transcription factors were found to be expressed in the larval photoreceptor cells at 72 h. These include *awh* (*arrowhead*; Lhx6/8-like), *foxg*, *hbn*, *nkx2.1*, *otx*, *six3*, *soxb2*, *tbx2/3*, and *zic* (Figs. 3A and 4). In addition, the expression of transcripts encoding the transcription factor Id was detected at 60 h and weakly also at 72 h in photoreceptors (Fig. 4). Many of these transcription factors are expressed in the apical organ bilaterally in just 1–3 cells in a pattern similar to Rx and opsin3.2, although some, such as Tbx2/3, are expressed more broadly in the oral/lateral region of the apical neurogenic ectoderm including the photoreceptors. The expression of *awh* and *six3* was furthermore analyzed by double-fluorescent WMISH together with probes detecting *opsin3.2*. The results showed that both genes are expressed in cells also expressing *opsin3.2*, confirming the expression in photoreceptor cells (Fig. S4). The regulatory state of these larval photoreceptor cells includes several transcription factors, such as Otx and Six3, in addition to Rx, that are also expressed in vertebrate eyes and photoreceptors.

To determine the developmental origin and timing of specification of the larval photoreceptors, the expression of regulatory and differentiation genes was analyzed at several stages during early sea urchin development. Based on transcriptome time course data, the expression of *opsin3.2* is initiated at 60 h, indicating that the differentiation of precursors into functional photoreceptor cells does not occur before 60 h (Fig. S1A) [35]. The developmental specification of photoreceptors during embryogenesis was determined by analyzing the expression of *rx* and other regulatory genes at 24, 36, 48, 60, and 72 h by WMISH (Fig. 4). The results show that *rx* is expressed broadly in the apical neurogenic domain at 24 h, as shown earlier [47]; however, expression in the oral/anterior region of the apical domain becomes restricted to the photoreceptors by 60 h. This expression pattern is reminiscent to the expression of *rx* in vertebrates. In mice, for instance, *rx* expression initiates in the anterior neural plate before becoming progressively restricted to the eyes and ventral forebrain, and later to photoreceptors [24]. Similarly, all transcription factors examined here are expressed in the oral apical domain by 36 h. Of these, *awh*, *nkx2.1*, *id*, and *six3* show specific expression in photoreceptors within the oral apical domain by 60 h, while the remaining transcription factors are expressed in broader areas including the photoreceptors (Fig. 4). Thus, a photoreceptor-specific regulatory state that is distinct from regulatory states expressed in the surrounding cells of the anterior neurogenic ectoderm is first identified at 60 h. Photoreceptor precursors originating in the anterior neurogenic domain thus become distinctly specified as photoreceptors by 60 h, which coincides with the onset of expression of *opsin3.2*. Based on the position and expression of transcription factors, these results suggest that the anterior neurogenic region provides the developmental origin of the larval photoreceptors.

### Expression of *pax6*, *six1/2*, *eya*, and *dach* in the hydropore canal but not in ciliary photoreceptors

**Fig. 5:**
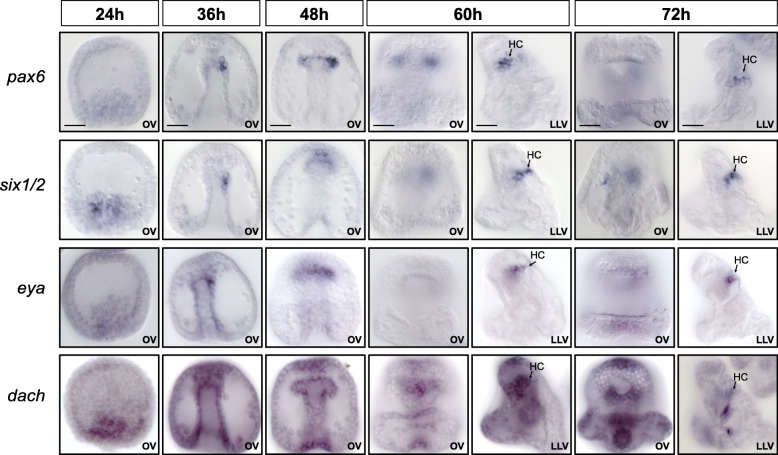
Developmental spatial expression of PSED regulatory genes. Images of embryos stained by WMISH for expression of four members of the retinal determination network at indicated developmental stages. Co-expression was observed in the hydropore canal but not in photoreceptor cells. HC, hydropore canal; LLV, left lateral view; OV, oral view. Scale bars 20 μm

Given their important role in eye development and in the specification of retinal progenitor cells throughout bilateria, we analyzed the expression of *pax6*, *six1/2*, *eya*, and *dach* (PSED genes) by WMISH (Fig. 3B). The expression of all four regulatory genes was detected in the hydropore canal, a mesodermal derivative for filtering and secretion of the coelomic fluid. However, the expression of these four regulatory genes was absent from larval photoreceptors at 72 h (Figs. 3B and 5) [48, 49]. To analyze whether the expression of these genes occurred during earlier stages of photoreceptor specification, the expression was analyzed at 24 h, 36 h, 48 h, and 60 h (Fig. 5). All four regulatory genes are expressed in the coelomic pouches during early gastrulation and show the expression in the hydropore canal starting at 60 h. However, expression of *pax6*, *six1/2*, *eya*, and *dac* was not observed in the photoreceptors or their precursors at any stage considered here. Our results thus indicate that the PSED module does not operate during the development or differentiation of the sea urchin larval photoreceptors, even though a functional PSED module is encoded in the genome and expressed in the hydropore canal.

## Discussion

The results presented here indicate that echinoderms possess ciliary photoreceptors that show similarities to the photoreceptors present in chordates. Thus, sea urchin larvae have photoreceptors that are positioned orally and bilaterally in the anterior nervous system just above the mouth. These photoreceptor cells are neurons, as indicated by the expression of synaptotagmin. Furthermore, the larval photoreceptors express opsins that are present on the cell surface cilia, and a set of transcription factors including Rx, Otx, and Six3, that are orthologs of transcription factors co-expressed in the eyes and ciliary photoreceptors in vertebrates. Taken together, these results lead to the conclusion that the larval photoreceptors correspond to ciliary photoreceptors that are specified by transcription factors that also contribute to the development of photoreceptors in vertebrate eyes. The association of these photoreceptors with pigment cells suggests that sea urchin larvae might be capable of directional light perception [4]. Indeed, the activity of the digestive tract in sea urchin larvae is regulated in response to light, by a mechanism depending on opsin3.2, which indicates that the photoreceptors are capable of initiating a behavioral response upon stimulation with light [34].

The presence of ciliary photoreceptors in sea urchin larvae indicates that sea urchins form both types of photoreceptor cells, ciliary and rhabdomeric photoreceptors. The tube feet of penta-radial adult sea urchins include rhabdomeric photoreceptors, based on their expression of opsin 4 and Pax6 [13, 32], and ciliary photoreceptors are present in bilateral larvae. Other basal deuterostomes including amphioxus, as well as protostomes such as *Platynereis* and several other species, also possess both types of photoreceptor.

### Evidence for homology of ciliary photoreceptors within deuterostomes

**Fig. 6:**
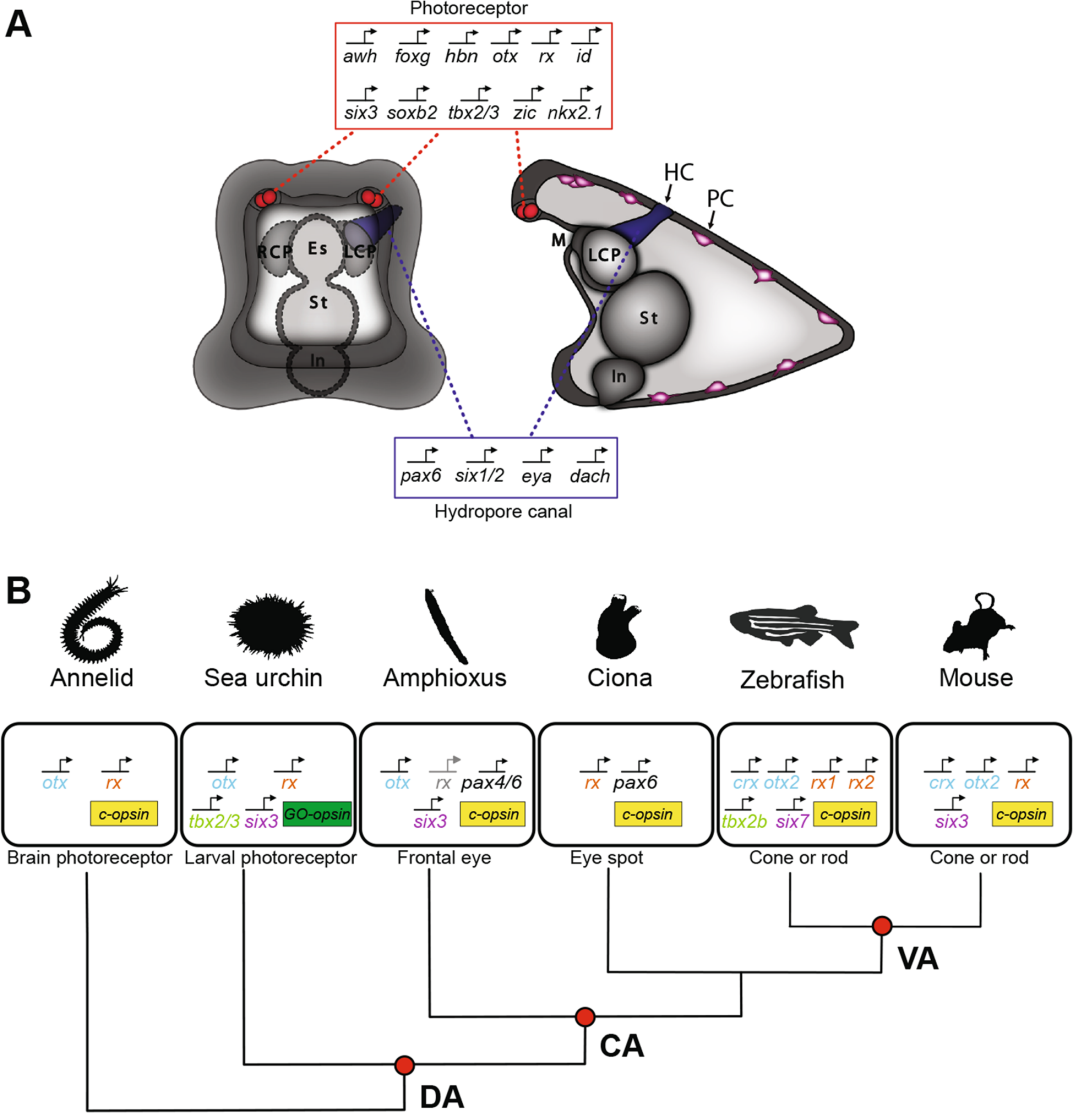
Summary diagram showing sea urchin photoreceptors and phylogenetic distribution of photoreceptor-specific regulatory states. **A** Summary diagram of sea urchin larva showing the expression of regulatory genes in photoreceptors and expression of indicated PSED genes in the hydropore canal (HC). Pigment cells (PC) are associated with photoreceptors and distributed throughout the aboral ectoderm. **B** Diagram showing the expression of a common set of regulatory genes in ciliary photoreceptors in animals representing specific positions within the phylogenetic tree. DA, deuterostome ancestor; CA, chordate ancestor; VA, vertebrate ancestor

Despite clear differences between echinoderm and chordate photoreceptors, and even of photoreceptors present within different vertebrate species, the present study revealed similarities which indicate that ciliary photoreceptors are homologous within deuterostomes. Thus, the specification of the sea urchin larval photoreceptors involves several transcription factors that are similarly expressed during the development of ciliary photoreceptors in other deuterostomes (Fig. 6A, B).

Most prominently, Rx is expressed in ciliary photoreceptors in many animals, including vertebrates, tunicates*,* amphioxus, and annelids [11, 21, 24, 29, 30, 50]. Sea urchin photoreceptors also express Otx, which is an ortholog of Otx2 and Crx that are necessary for photoreceptor specification and differentiation in vertebrates [18]. Otx is also involved in the photoreceptor specification of amphioxus [11] and brachiopods [51]. Comparable to sea urchins, Six3 is broadly expressed in the anterior neural plate region including photoreceptors of amphioxus [11], while its ortholog Six7 is required in zebrafish for the development of photoreceptors and the regulation of opsin expression [52, 53]. Six3 is also expressed in the mouse retina and has been associated with the regulation of rhodopsin expression [54]. Furthermore, members of the Tbx2 family are involved in the specification of UV cone cells in the zebrafish retina [55] and in the specification of the eye field in Xenopus [28]. The forkhead transcription factor FoxG is involved in the development of the telencephalon in vertebrates and expressed in the developing eyes in zebrafish and mice [56]. In sea urchins, *foxg* is expressed throughout the anterior neurogenic ectoderm including cells giving rise to the larval photoreceptors. The bHLH transcription factor Id is expressed during the initial specification of sea urchin photoreceptors at 60 h, but expression starts to fade and is barely detectable at 72 h. Similarly, in vertebrates, orthologs of Id transcription factors are expressed in retinal progenitor cells in zebrafish and mice and are downregulated in differentiating photoreceptors [57, 58]. Finally, orthologs of the zinc finger transcription factor Zic that in sea urchins is expressed early on in the precursors of photoreceptor starting at 36 h are expressed during mouse development in retinal progenitor cells [59]. As summarized in Fig. 6B, these data indicate that a majority of transcription factors expressed during the development of the sea urchin larval photoreceptors are commonly used for the specification and differentiation of ciliary photoreceptors within deuterostomes.

Based on gene expression, the sea urchin larval photoreceptor cells developmentally originate from precursor cells in the anterior/oral region of the apical neurogenic ectoderm. The developmental expression of *rx*, for example, occurs at first broadly in the anterior neural ectoderm, and later becomes progressively restricted to the photoreceptor cells in sea urchin larvae, comparable to the developmental trajectory of the *rx* expression in many vertebrate species [21]. The developmental origin of sea urchin larval photoreceptors is also reminiscent of the neural ectodermal origin of photoreceptors in *Ciona* and amphioxus [60].

Taken together, the similarities in embryonic origin and in the expression of cell type-specific combinations of transcription factors suggest that the sea urchin ciliary photoreceptors have not evolved independently. Instead, echinoderm and chordate ciliary photoreceptors are likely to have evolved from a common evolutionary origin and thus represent a cell type that is homologous at least within deuterostomes.

### Expression of an atypical opsin in sea urchin photoreceptors

Ciliary photoreceptors in chordates and also in a few protostome species typically express c-opsins, and opsin expression has been traditionally used as a marker for photoreceptor cell type identity. The sea urchin ciliary photoreceptors on the other hand express a different type of opsin, a Go opsin (Fig. S2). However, recent molecular data show that opsin gene expression has been repeatedly co-opted during the evolution of photoreceptor cells, sometimes leading to the co-expression of different types of opsin within the same photoreceptor cells [8, 18, 61]. Thus, the expression of particular opsins is not necessarily a reliable indicator of photoreceptor cell type identity. Members of the Go family of opsins have been found to be expressed in photoreceptors of several marine protostome species [61–63]. Ciliary photoreceptors of the distal retina of the bay scallop *Pecten irradians* for instance have been shown to use Go opsin as the primary photopigment which activates a light-response pathway that is different from the pathways activated by c-opsins and r-opsins [4, 38, 62]. Go opsins are also deployed in the photoreceptors of *Platynereis*, where opsin1 has been shown to be sensitive to cyan light at wavelengths important for marine life [39].

Although it cannot be excluded at this point that the Go opsin expressing photoreceptors of sea urchin larvae evolved independently from the chordate ciliary photoreceptors, a random co-option of almost the entire gene regulatory network responsible for photoreceptor specification represents an unlikely scenario. Instead, it appears more likely that ancestral photoreceptors present in deuterostome ancestors, expressing either c-opsins or Go opsins and their respective phototransduction machinery, would have given rise to echinoderms and chordate photoreceptors that are specified by a set of conserved transcription factors while evolutionary changes occurred in the expression of effector genes. Similar evolutionary scenarios have been observed in the immune system of deuterostomes, where conserved transcription factors regulate the expression of different immune effector genes [1, 64]. Thus, the expression of similar transcription factors might serve as a better indicator of cell type conservation than effector genes that continuously evolve to fulfill specific physiological and behavioral functions.

### Pax6 and the evolutionary history of eyes and ciliary photoreceptors

Transcription factors related to Pax6, Six1/2, Eya, and Dach (PSED) are involved in eye development in *Drosophila*, vertebrates, and many other animals [65]. The important role of Pax6 during eye development has been demonstrated in many animals where mutation of *pax6* typically leads to severe effects in eye development. The extraordinary similarity in the requirement of Pax6 during early eye development in distantly related animals including *Drosophila* [66] and vertebrates [67], and the ability of Pax6 to induce ectopic eyes when overexpressed in ectopic locations [68, 69], has led to the hypothesis that Pax6 and several other transcription factors of the retinal determination network function as “eye master regulators” that were expressed in the proto-eyes of bilaterian ancestors [16]. The question therefore arises whether the expression of a shared set of transcription factors in the eyes of flies and vertebrates is a result of evolutionary conservation and already present in bilaterian ancestors, or a result of evolutionary co-options of PSED regulatory circuits during the evolution of complex eyes [6].

The absence of PSED transcription factors in sea urchin larval photoreceptors might be the result of an evolutionary loss of function that occurred in the echinoderm lineage but on the other hand might also represent the ancestral state of simple eyes in deuterostome ancestors. To reconstruct the most likely ancestral state of ciliary photoreceptors, a comparison with molecular data from other deuterostomes is informative. Pax6 for example, although not expressed in sea urchin embryos, is expressed in the ciliary photoreceptors of invertebrate chordates such as amphioxus and *Ciona* [11, 70]. Pax6 is also expressed in the apical neurogenic ectoderm of sea star larvae, although photoreceptors have not been described in this species [71]. In vertebrates, Pax6 is specifically required during early eye development and contributes to the maintenance of multi-potency in retinal progenitor cells [67, 72]. However, during later stages of retinal development, Pax6 controls the specification of horizontal and amacrine cells and inhibits the differentiation of ciliary photoreceptor cells [73]. Thus, Pax6 functions in the progenitors of photoreceptors but not in differentiated ciliary photoreceptors. This is consistent with the observation that the expression of differentiation genes such as *c-opsins* does not require Pax6 in vertebrate ciliary photoreceptors but instead is controlled by Otx and Rx [5, 21]. The absence of Pax6 function is therefore a shared feature of ciliary photoreceptors in echinoderms and vertebrates, indicating that Pax6 does not constitute a conserved component of the cell type-specific regulatory state in differentiated ciliary photoreceptors.

The expression of other PSED transcription factors has been analyzed in detail in amphioxus, showing that PSED factors are co-expressed in many cell types but not in ciliary photoreceptors [74]. And while several PSED transcription factors are expressed during early eye development in vertebrates, their function, in particular during the differentiation of photoreceptors, is not as clearly resolved as in *Drosophila* [5, 6]. The absence of PSED transcription factor expression in ciliary photoreceptors of sea urchin and amphioxus suggests that the PSED module might not have been required for the specification of ciliary photoreceptors in early deuterostomes and might have been co-opted for the specification of retinal progenitors during the evolution of complex vertebrate eyes.

Consistent with the idea that PSED transcription factors might have been co-opted to eye development, PSED genes have been co-opted as a module to many other developmental processes. Thus, in vertebrates, PSED factors contribute to kidney development and the specification of somitic muscle [75], and in amphioxus, PSED factors are co-expressed in several cell fates other than photoreceptors [74]. Similarly, PSED genes are expressed in coelomic pouches of *Lytechinus variegatus* sea urchins [49] and, as we show here, in the larval hydropore canal in *S. purpuratus*. These observations suggest that the PSED module controls functions that are required in many different developmental contexts and thus has been co-opted frequently, including into the gene regulatory network controlling early eye development in vertebrates [7].

## Conclusions

The evidence presented here suggests that ciliary photoreceptors are present in the larva of echinoderm sea urchins and based on developmental and molecular similarities are homologous to ciliary photoreceptors of chordates despite substantial differences. This would indicate that the ancestry of ciliary photoreceptor cell types dates back at least to the origin of deuterostomes. Subsequent evolutionary modifications in the gene regulatory network controlling eye development, possibly including the co-option of the PSED module, would have given rise to the variety of photoreceptors and eyes within deuterostomes.

## Methods

### Phylogenetic analysis and identification of orthologs

Opsin dataset was obtained by merging the sequences from [39] and [36]. Furthermore, additional opsin genes were obtained from the genomes of *Branchiostoma floridae* [76], *Branchiostome belcheri* [77], *Ciona intestinalis* [78], and *Ciona savignyi* [79]. Specifically, the dataset of [37] composed of 449 sequences was used as seed and potential homologs were identified using BLASTP [80]. Each sequence with an *e*-value < 10^−10^ was retained a good opsin homolog. To identify opsin genes, sequences were further annotated using interproscan [81], and only sequences with retinal binding domains were considered as Opsins. The final dataset includes 232 Opsins and 10 melatonin receptors that have been used to root the trees. Alignment was performed using MAFFT [82], and phylogenetic reconstruction was performed under the maximum likelihood framework and Bayesian framework under LG-G_4_ [83]. The ML tree was reconstructed using iqtree [84], and nodal support was estimated using ultrafast bootstrap [85] (1000 replicates) and the SH-aLTR bootstrap [86]. Bayesian inference was performed using Phylobayes4.1 [87] with two independent runs. Convergence was evaluated using tracecomp and bpcomp packages in Phylobayes (see Phylobayes manual). Alignment and trees are available at https://github.com/RobertoFeu/Opsins_phylogeny_Valencia_et_al. The identification of orthologs in Fig. 6B was performed using EggNOG mapper [88].

### Gene amplification and probe synthesis

The primer sets used for gene amplification are listed in Table S1. Gene models generated from sea urchin transcriptome analysis were used as a reference for primer design [35] using T7 tailed primers or cloning. cDNA prepared from various developmental stages was used as a template for PCR. For cloning, PCR products were purified and ligated into GEM-T EZ constructs. Cloned genes were PCR-amplified using the primer flanking the insert region, and PCR products were used to synthesize RNA probes for WMISH.

### Whole-mount in situ hybridization

The protocol for whole-mount in situ hybridization (WMISH) to detect spatial gene expression has been described previously [89]. Briefly, sea urchin embryos were fixed in 4% paraformaldehyde solution. The fixed embryos were incubated in hybridization buffer [50% (vol/vol) formamide, 5× SSC, 1× Denhardt’s, 1 mg/mL yeast tRNA, 50 ng/mL heparin, and 0.1% Tween-20] with a concentration from 1 to 2 ng/μL digoxygenin RNA probe(s) at 60 °C for 18 h. Two post-hybridization washes were performed with hybridization buffer without RNA probe, 2× SSCT (2× SSC, 0.1% Tween-20), 0.2× SSCT, and 0.1× SSCT, each 20 min at 60 °C. Subsequently, 3 washes were performed with MABT buffer (0.1 M maleic acid, 0.15 M NaCl, and 0.1% Tween-20). Antibody incubations were performed at room temperature with 1:2000 diluted anti-DIG Fab (Roche). The embryos were washed with MABT buffer and with AP buffer (100 mM Tris·Cl (pH 9.5), 100 mM NaCl, 50 mM MgCl2, and 1 mM levamisole). 5-Bromo-4-chloro-3-indolyl-phosphate (BCIP) and nitro blue tetrazolium were used for staining. Fluorescent in situ hybridization protocol was performed as described in [90].

### Antibody production

Antibody production was as previously described [91]. Antigens were made using a pET28b (+) plasmid (Novagen) for the expression of tagged proteins. An Opsin3.2 construct was prepared by PCR amplification (opsin-cyto:F = 5′-CAGTCATATGGCGTCGGTAAAATAAG-3′, opsin-cyto:R = 5′-AGTCAAGCTTCTGTAGATTTTTAATG-3′) encoding 844–1494 bp of the coding sequence and 282-498aa of the protein. This opsin DNA fragment was amplified from *S. purpuratus* cDNA and cloned into the pGEM-T Easy system (Promega). Protein expression was induced in *Escherichia coli*. Purified protein in PBS was mixed 1:1 with Freund’s adjuvant for immunization. Rats were immunized by subcutaneous injection of 100 mg antigen in 250 μL of adjuvant, and booster injections were done 21 days and 42 days after the initial immunization. Blood was collected after 52–56 days and centrifuged to collect serum. Antigen specificity of polyclonal antibodies was tested by pre-absorbing the immune serum with an approximately equimolar preparation of the protein used to inoculate the rat. Pre-absorption eliminated antibody binding to 72- and 96-h larvae, confirming antibody specificity.

### Immunofluorescence

*S. purpuratus* embryos were collected at the desired time point and fixed for 5–10 min in 4% paraformaldehyde in PEM buffer [92]. Embryos were washed with phosphate-buffered saline (PBS), blocked for 1 h in SuperBlock (Thermo), probed with primary antibody, and washed 3 times with PBS. Alexa Fluor fluorescent secondary antibodies (Invitrogen) were used to visualize antibody labeling on a Zeiss 700 LSM (Carl Zeiss) confocal microscope. All preparations were done at 4 °C. Imaging and analysis were conducted using ZEN (2009) or ImageJ (1.44) software. Adobe Photoshop (9.0.2) was used to prepare the figures and adjust image contrast and brightness. Antibodies deployed here: anti-SynB [43], Sp1 [46], and anti α-tubulin (Santa Cruz Biotechnologies, sc-23948).

## Supplementary Information

**Additional file 1: Fig. S1.** Expression of opsin genes. **Fig. S2.** Phylogenetic analysis of opsins. **Fig. S3.** Expression of synaptotagmin. **Fig. S4.** Expression of additional regulatory genes. **Table S1.** Primer sequences.

## Data Availability

All data generated during this study are included in this published article [and its supplementary information files]. Alignments of opsins are available at https://github.com/RobertoFeu/Opsins_phylogeny_Valencia_et_al.
