# Supplementary material for: Ciliary photoreceptors in sea urchin larvae indicate pan-deuterostome cell type conservation
Source: BMC Biol. 2021 Dec 4;19:257. doi: 10.1186/s12915-021-01194-y (PMC8642985; doi:10.1186/s12915-021-01194-y)
Supplement: Supplementary file 1 — Additional file 1: Fig. S1. Expression of opsin genes. Fig. S2. Phylogenetic analysis of opsins. Fig. S3. Expression of synaptotagmin. Fig. S4. Expression of additional regulatory genes. Table S1. Primer sequences. [file 12915_2021_1194_MOESM1_ESM.pdf]

ADDITIONAL FILE 1

Ciliary photoreceptors in sea urchin larvae indicate pan-deuterostome cell type conservation

Jonathan E. Valencia[#,1], Roberto Feuda[#,1,2], Dan O. Mellott[3], Robert D. Burke[3,*], Isabelle S. Peter[1,*]

[1] Division of Biology and Biological Engineering, MC156-29, California Institute of Technology, Pasadena, California 91125

[2] Present address: School of Earth Sciences, University of Bristol, Bristol, UK

[3] Department of Biochemistry and Microbiology, University of Victoria, Victoria, British Columbia, Canada

# equal contribution

* Corresponding authors. Email: ipeter@caltech.edu (ISP); rburke@uvic.ca (RDB)

Figure S1

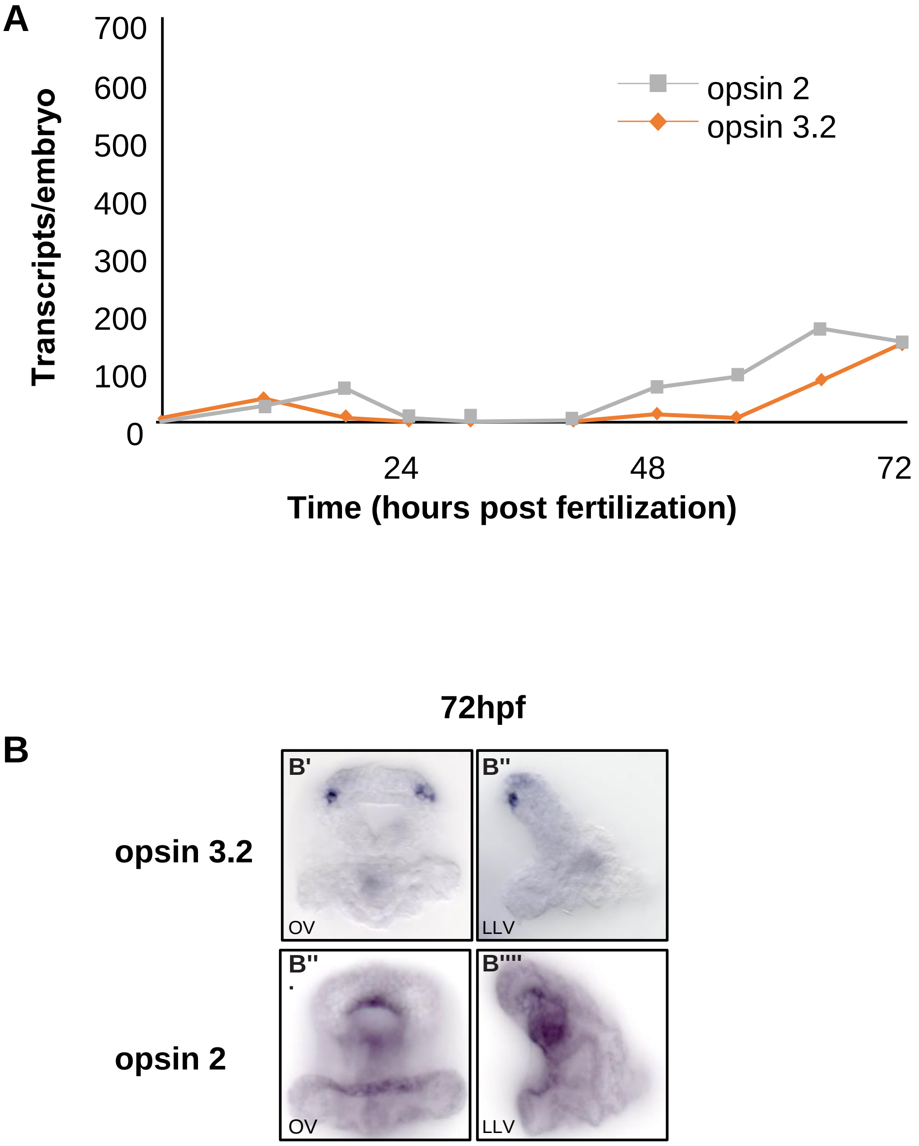

Figure S1. Expression of opsin genes in putative photoreceptors in sea urchin larvae. **(A)** Developmental time course of opsin gene expression based on transcriptome data from Tu et al 2014. **(B)** Spatial expression of opsin 3.2 and opsin 2 in 72h sea urchin larvae. OV, oral view; LLV, left lateral view.

Figure S2

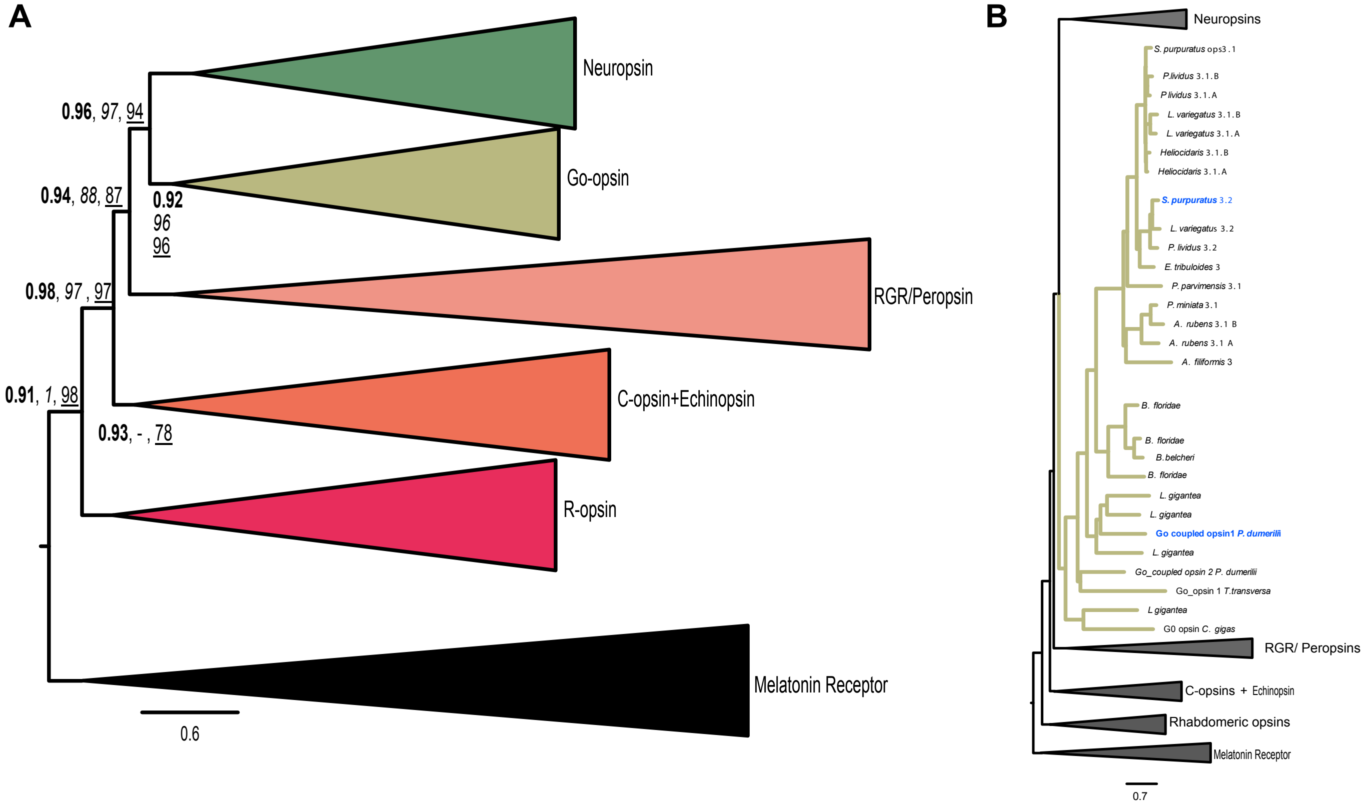

Figure S2. Phylogenetic analysis of opsins. (A) Phylogenetic tree of opsins. Values in bold indicate the Bayesian posterior probability, values in italics indicate SH-aLTR bootstrap values, values underlined indicate ultrafast bootstrap (1000 replicates). Major opsin clades are color coded. The three group topology where R-opsins are a sister group of C-opsins and RGR/Go-opsins is well supported. **(B)** Phylogenetic tree as in (A) with focus on Go-opsins. Opsin 3.2 is a co-ortholog of Go-opsin1 of *Platynereis dumerilii*, which was recently shown to be sensitive to cyan blue light.

Figure S3

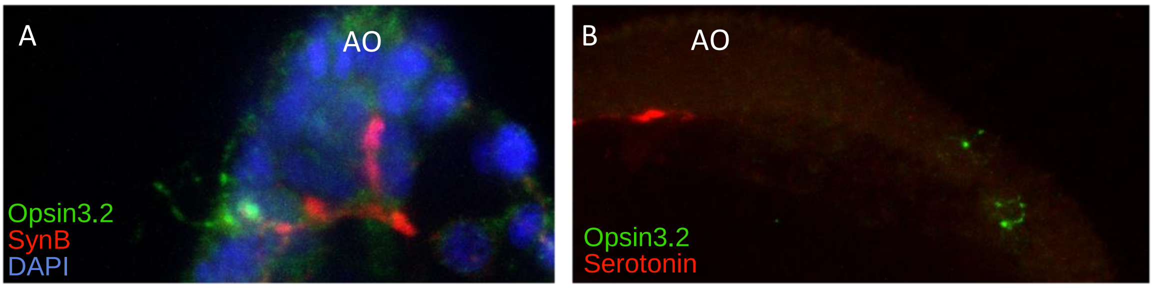

Fig. S3. Expression of synaptotagmin but not serotonin in photoreceptors. (A) Co-localization of Opsin3.2 and SynaptotagminB. Confocal projection from a left lateral perspective showing the projection of a SynaptotagminB containing axon into the basal neuropil of the Apical Organ (AO). **(B)** Co-immunostaining of Opsin3.2 and Serotonin indicates that PRCs do not contain serotonin. The apical organ is known to include serotonergic neurons on the dorsal margin, and the photoreceptor cells project axons into the apical organ, but do not contain Serotonin.

Figure S4

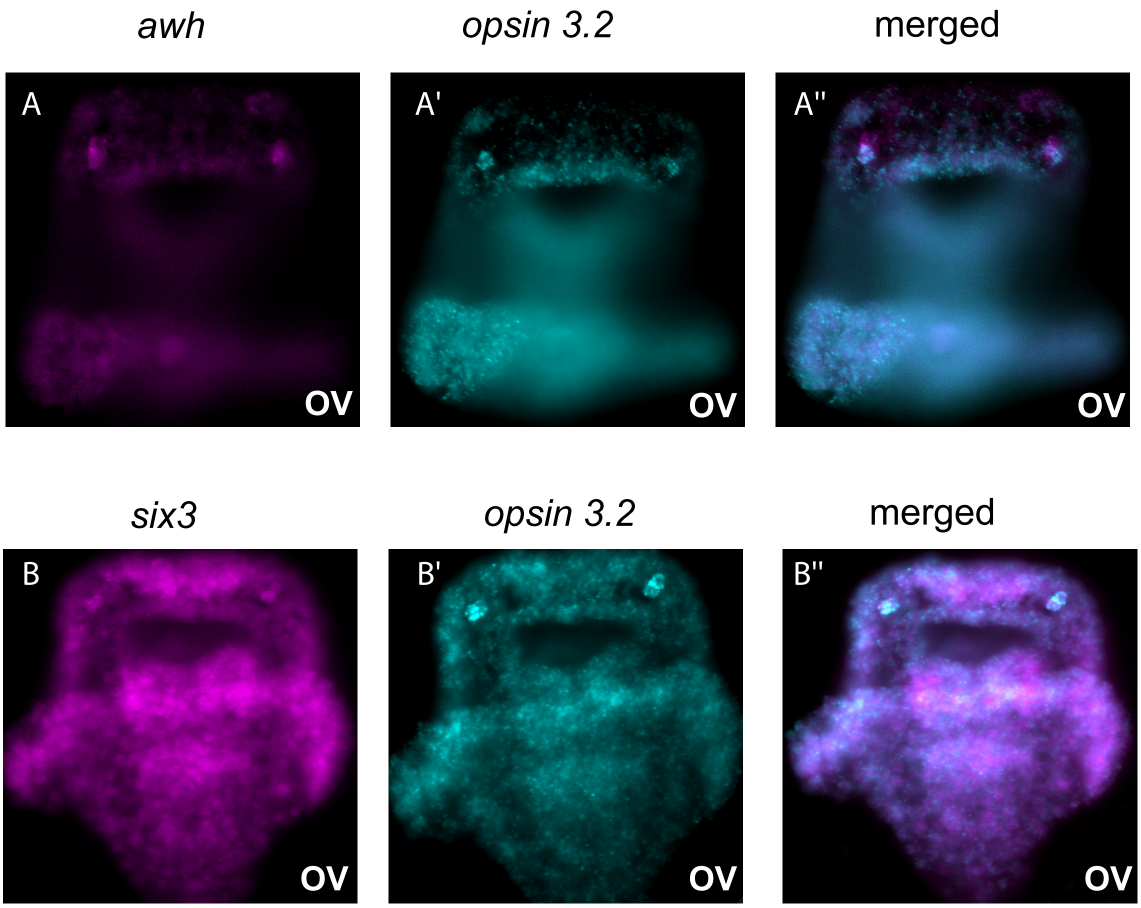

Fig. S4. Expression of additional regulatory genes in PRCs. Double fluorescent WMISH of *opsin3.2* and (**A**) *awh* or (**B**) *six3* confirming expression in PRCs. OV, oral view

Table S1

Gene name	Transcript ID	Forward	Reverse
Awh	WHL22.9369.1	CATAACCATCCCATCAATAAATCC	TAATACGACTCACTATAGGGAGATGCACAGCACTCTATTTTCAATC
Dac	WHL22.169355	GATGCGAACCTGTTCTACG	TAATACGACTCACTATAGGGAGACAATTCAAAAGCTTGTGGCA
Eya	WHL22.168736	GTATTGGAAGAGGGCGTCAA	TAATACGACTCACTATAGGGAGAATGACTTGTTACCCGCCAG
FoxG	WHL22.389872.0	GCGCTTTACTCGTCTTATTCTACC	GTCCTTAGTTGAAATGGGAAACC
Hbn	WHL22.523959.1	TCATTACTTCGTTGGAGTTACCC	CATGAAAACGTCTGGATACTGG
Ia1	WHL22.769122.0	ACCCTACAAGTGCAACTGAAACA	ATGGGCAAGTTGTGCAGTAATAA
Id	WHL22.467043	CGTCCTAATTTTAACGTGTTTGG	AATATGTCTTTCGGCGTTGTAGA
Nkx2.1	WHL22.739581.0	AAGCAGCAGAAGTACCTGTCG	TAATACGACTCACTATAGGGAGAATGACTATTGTGTGGTGCAAGC
Opsin2	WHL22.272775	CGTTAATGTCCCATGCTGTG	TAATACGACTCACTATAGGGAGACTTTGGGCAAGACAGCAGAT
Opsin3.2	WHL22.338995	CGGTAACATCACCGTCCTTT	TAATACGACTCACTATAGGGAGACGGAATTTGGAGCTTGATGT
Otx	WHL22.532435.1	AACAGCAGCAACAGCAACAG	AGAGCTGCGTTCAAGGTCAT
Pax6	WHL22.585512.0	CGCAATCAGAGAAGACAGCA	TTAGCCAGCAAGAAGGGAAA
Rx	WHL22.523971.0	AAGAGCAACGGTGGAATAAAAAC	GCTGATTATACGTTCAGGCAAGA
Six1/2	WHL22.121485.0	GCAATAACTTCTCACCGCATAAC	TAATACGACTCACTATAGGGAGAGTTCATGTTTTTCTTTCCGACTG
Six3	WHL22.121654.0	CTCATAGACACACCCCAGCA	AGGATGGTGGGATCTTTCTTC
SoxB2	WHL22.104525.0	ATCAGAGACTTTCCCCATCATC	TAATACGACTCACTATAGGGAGAGTGTTGCACAGTCCTTGTTGAC
SoxC	WHL22.622787.0	GTCACAAATCGAGAGGAGACG	TAATACGACTCACTATAGGGAGATCTGAGTCTATGAGTTCGCTTACC
Tbx2/3	WHL22.457020.0	TCACAAAAGAGGAACAGAAATGG	TAATACGACTCACTATAGGGAGAGGGATGGGTGTCTAAATAACTCG
Zic	WHL22.331651.0	CAATCGCGTTTCAGTTGACTAC	ACGTACCATTCACTCAAGTTCGT

Table S1. Primers used for generation of WMISH probes. The transcript ID refers to the transcriptome-based gene model defined in [1]. Gene sequences can be found on Echinobase (http://www.echinobase.org/Echinobase/).
